# “Losing the Phobia:” Understanding How HIV Pre-exposure Prophylaxis Facilitates Bridging the Serodivide Among Men Who Have Sex With Men

**DOI:** 10.3389/fpubh.2018.00250

**Published:** 2018-09-06

**Authors:** Kimberly A. Koester, Xavier A. Erguera, Mi-Suk Kang Dufour, Ifeoma Udoh, Jeffrey H. Burack, Robert M. Grant, Janet J. Myers

**Affiliations:** ^1^Division of Prevention Science, Center for AIDS Prevention Research, School of Medicine, University of California, San Francisco, San Francisco, CA, United States; ^2^East Bay AIDS Center, Sutter Health, Oakland, CA, United States; ^3^Virology and Immunology, Gladstone Institutes, University of California, San Francisco, San Francisco, CA, United States

**Keywords:** HIV prevention, HIV serodivide, PrEP, pre-exposure prophylaxis, HIV stigma, serosorting, seromixing, MSM

## Abstract

The use of HIV serostatus information has played a pivotal role in partner selection norms. A phenomenon known as *serosorting* is the practice of selecting a partner based on a perception that they are of the *same* HIV status in order to avoid transmission from one partner to the other. An understudied aspect of serosorting is that it has a divisive effect—one accepts or rejects a potential partner based on a singular characteristic, the partner's HIV status, and thus excludes all others. This division has been formally referred to as the HIV *serodivide*. In this study, we explored partner selection strategies among a group of HIV-negative, young men who have sex with men (*n* = 29) enrolled in a PrEP demonstration project in Northern California. We found that trends in serosorting were in fact shifting, and that a new and opposite phenomenon was emerging, something we labeled “seromixing” and that PrEP use played a part in why norms were changing. We present three orientations in this regard: (1) *maintaining the phobia*: in which men justified the continued vigilance and exclusion of people living with HIV as viable sex or romantic partners, (2) *loosening/relaxation of phobia*: among men who were reflecting on their stance on serosorting and its implications for future sexual and/or romantic partnerships, and (3) *losing the phobi*a: among men letting go of serosorting practices and reducing sentiments of HIV-related stigma. The majority of participants spoke of changing or changed attitudes about intentionally accepting rather than rejecting a person living with HIV as a sex partner. For those who maintained strict serosorting practices, their understandings of HIV risk were not erased as a result of PrEP use. These overarching themes help explain how PrEP use is contributing to a closing of the HIV serodivide.

## Introduction

Romantic and sexual partnerships fluctuate over time and depend on opportunities as well as values held at the individual and societal level. In the United States, partner selection norms within communities of gay and other men who have sex with men (G/MSM) have been shaped in different ways depending on how men relate to the social construction of HIV. Some men perceive HIV as an illness that bonds the gay community; others, as an illness that brings shame to the gay community. The medical response to HIV i.e., technologies designed to prevent and manage HIV disease (e.g., anti-retroviral therapies, HIV testing) and the belief in their efficacy, influence partner selection, and sexual norms as well.

The use of HIV serostatus information among G/MSM has played a pivotal role in partner selection norms ranging from *including* anyone as a potential partner regardless of HIV status to *excluding* anyone of serodiscordant status (1). According to Race, prior to the advent of serosorting, members of the gay community were determined to include rather than exclude men living with HIV as viable partners; condoms enabled full participation in sexual worlds regardless of one's HIV status. Eventually alternative practices emerged. A number of scholars e.g., Kippax (2), Race (1), Halperin (3) have found that gay men's sexual practices continually evolve as the science of HIV/AIDS treatment and prevention advances. One key strategy that developed among gay men was the use of HIV status information in sexual decision making. We examine this and other *seroadaptive* strategies in more detail later, including routine HIV screening, strategic positioning, and withdrawal prior to ejaculation (2, 4). In this paper we focus on the risk reduction strategy known as *serosorting*- the practice of selecting a partner based on a perception that they are of the *same* HIV status, in order to avoid HIV transmission from one partner to the other (5) and in many cases to forego the use of condoms altogether (1, 6).

Knowing one's HIV status is a critical first step to serosorting, though serosorting can and does happen in the absence of this information (7, 8). Using knowledge of one's HIV status to effectively serosort is a practice fraught with micro-dilemmas (9) and is tied to contextual factors (4). For example, it assumes that individuals are able and willing to communicate about HIV status and that individuals are honest and accurate regarding disclosure of their status. A study of HIV-positive gay and bisexual men found that 42% reported any sex (either with or without condoms) without disclosing their status (10). While serosorting is not limited to G/MSM by any means, it has been one of many important harm reduction strategies practiced for years among G/MSM (11–13).

HIV testing is an important element to effective serosorting and access to such technology is unevenly distributed throughout the globe. In the United States, clinic-based HIV testing is paid for by public and private insurance and can be obtained for free through mobile testing units and stand-alone testing sites. HIV home test kits can be purchased in the United States in pharmacies or online for as little as 31 US dollars. Yet 54% of the general population has ever been tested and rates of routine testing among G/MSM occurs only among ~1 in 5 (14). HIV-related stigma, medical mistrust and fear prevent people from testing at all or testing on a consistent basis (15).

One under-acknowledged byproduct of the practice of serosorting is that it is inherently reductionist; one accepts or rejects a potential partner based on a singular characteristic—their HIV status. The roots of this division into the binary categories of HIV-positive or HIV-negative emerged in 1995 facilitated by improvements in the HIV testing technology (16). Though in the social science literature (1, 17–19) serosorting is largely responsible for fostering the HIV serodivide—a phenomenon whereby persons avoid having sex with others of different HIV status despite having access to prevention tools, i.e., condoms and strategic positioning—that would enhance safer sex. While the concept of the HIV serodivide is not used in everyday discourse, the implied meaning resonates with anyone who has been rebuffed by a prospective partner because of his or her HIV status.

Tensions and harms caused by the HIV serodivide are not limited to those who are HIV-infected. People who are HIV-negative and partnered with a person living with HIV (PLWH; in a serodiscordant relationship) may suffer from a version of vicarious stigma (witnessing or hearing about HIV-related stigma which then becomes internalized) (20, 21). G/MSM who are HIV-uninfected and highly risk averse may contend with feelings of ambivalence and shame related to their practice of excluding PLHW as partners (1). However, the effect of exclusion is most pronounced for those who are living with HIV (22). Many PLWH struggle with feelings of rejection (23), shame (24), and low sexual self-esteem (25). While a person living with HIV may have these feelings soon after being diagnosed, some PLWH may continue to face these difficult feelings for years (26) while others do not (27). Feelings of rejection, shame and low sexual self-esteem are emotional states likely stemming from perceived or felt HIV-related stigma (28). To avoid experiencing such emotional states, some people living with HIV opt to sexually partner with other people living with HIV a practice that maintains one side of the serodivide (29).

In this paper, we examine changes in partner selection practices in the context of PrEP use. Our data add to a growing body of evidence that suggests a more intact and cohesive community may be underway with greater uptake of highly effective biobehavioral prevention approaches that include treatment as prevention (TasP) and PrEP. Like TasP, PrEP is a highly effective HIV prevention strategy, the uptake of which continues to grow since its regulatory approval occurred in 2012, particularly among G/MSM in urban areas (30, 31) of the US. First we review the historical emergence of seroadaptive partnership strategies, with a particular focus on serosorting. Then we discuss the shifting sexual, emotional, and communicative benefits described by gay men when employing these strategies to reduce the risk of HIV transmission. Finally, we illustrate contemporary occurrences of sexual partnering among a cohort of young G/MSM PrEP users living in an urban city outside of San Francisco, California a location with a history of robust access to HIV prevention and care services. We describe and characterize how participants made sense of HIV risk in the context of PrEP use to better understand when and in what context PrEP use engendered shifts in attitudes about HIV. We hypothesize that these shifts may ultimately contribute to decreased HIV-related stigma in the gay community.

## Literature review

HIV prevention strategies are not static and change over time and so does their influence on partner selection practices. Diverse strategies intended to reduce HIV transmission and acquisition have emerged from communities most impacted by the epidemic (12, 13, 32, 33). Over the last 20 years, use of HIV serostatus to inform decisions around sexual behavior, including partner selection and risk negotiation, has become an important area of research [see for example, (5, 8, 34–39). The earliest investigations attempted to inform HIV transmission dynamics by modeling partnership selection patterns prior to the wide availability of highly-active antiretroviral therapy [HAART; see for example, (40–45)]. In the absence of empirical data, these early investigations often relied on theoretical models and *a priori* assumptions.

Building on this early research, investigators used survey data from large cohorts of ostensibly “high-risk” individuals to understand the impact of partner selection on transmission dynamics. This allowed prevention scientists to investigate the relationships between self-reported sexual behavior and sexual health outcomes of interest. These efforts sought to demonstrate the utility of tracking biobehavioral markers to forecast shifts in the HIV epidemic, such as increases in condomless sex, rates of sexually transmitted infections and HIV disclosure rates (46–51). These survey studies found people reported higher HIV risk behaviors after HAART became widely available. This led some researchers to attribute the observed increase in self-reported HIV risk behaviors to treatment optimism, being overly optimistic about the benefits of HIV treatments [(32, 52–55)] and prevention fatigue, the sense that prevention messages have become tiresome (56–62). These studies implied that individuals who had sex with multiple partners and did not regularly use condoms during sexual encounters were either unconcerned about HIV and/or unreceptive to prevention messaging.

One major limitation of these early surveillance studies is the fact that self-reported sexual behavior was being compared against a static understanding of HIV prevention. In focusing on individual sexual behaviors, these studies restricted the scope of prevention strategies to those one can implement by themselves, such as abstinence, reduction in numbers of partners, avoiding anal sex, and condom use (46, 48, 49, 51, 63–66). These surveys did not allow for individuals to report the use of seroadaptive strategies, such as serosorting, which depend on interactional and dyadic factors like HIV disclosure and partnership selection. This resulted in seroadaptive prevention strategies being under-acknowledged as protective and often misidentified as sexual disinhibition (50, 51, 66–68). Nonetheless, these studies were instrumental in demonstrating the widespread prevalence and increasing reliance on seroadaptation strategies for HIV risk reduction, by both people infected and uninfected with HIV (8, 39, 69–75).

The efficacy of seroadaptive strategies became a focus of ardent discussion when puzzling data from San Francisco showed that, between 1993 and 1999, decreases in condom use and a spike in STI rates among gay and bisexual men *did not* result in increased HIV incidence over the same time period (6, 50, 51, 74, 76, 77). These findings turned on its head widely held assumptions concerning key populations' attitudes and beliefs about HIV risk and prevention (12, 13, 76–80). Investigations to understand when and for whom seroadaptation strategies are effective have resulted in a large body of research on the sexual practices among priority populations. Through these investigations, we have gained a more nuanced understanding about the dynamics of seroadaptation strategies.

We know that the effectiveness of seroadaptation strategies depends on a number of key elements, such as knowing one's HIV status (79, 81, 82), frequency of HIV testing (38), and efficacy with HIV disclosure (8, 37, 83, 84). Seroadaptation strategies are sensitive to various individual, dyadic and community level factors, including race (85), and ethnicity (86–88), partnership types (89, 90), ability to detect early infections (13), and the prevalence of undiagnosed HIV in a community (38, 91). Advancements in HIV testing technology have ostensibly made it easier to provide HIV testing in a wider range of settings and to reach more people with undiagnosed HIV infection (92–94). However, stigma related to HIV continues to hinder the potential preventative benefits of seroadaptive strategies by delaying testing and making disclosure less likely (17, 24, 95).

The emergence of PrEP and TasP, two highly effective bio-behavioral HIV prevention strategies that work with or without concurrent condom use, present an opportunity to consider the social ramifications of certain frameworks for understanding sexuality and risk and the consequences these frames have in perpetuating the separation of people based on serostatus (33, 80, 96–104). However, self-imposed restrictions on sex and dating between people infected with or uninfected with HIV may be diminishing. Persson and colleagues documented the legitimizing effects that relying on antiretroviral therapy to prevent onward transmission (TASP) is having on gay and heterosexual serodiscordant relationships (19, 105). Other researchers call attention to the diversity of HIV status identity options emerging in G/MSM social media and dating platforms e.g., “undetectable” or “on PrEP” rather than the binary labels of “poz” or “neg.” These more nuanced additional identities suggest disruptions to the otherwise simplistic HIV serodivide anchored by two opposing points (18).

Our analysis seeks to contribute to the social science literature by offering contemporary examples of seroadaptive strategies deployed by young gay and other men who have sex with men in Northern California in an era of increasing uptake of HIV pre-exposure prophylaxis. This study took place during a time of major transition in HIV prevention and provides insight into a growing sense of cohesion among communities of people infected and affected by HIV.

## Methods

Data for this study come from a multi-year demonstration project, Connecting Resources for Urban Sexual Health (CRUSH), funded to test innovative approaches to improve sexual health outcomes among adolescents and young adults at risk for or living with HIV with the ultimate goal of curbing the HIV epidemic in California (106, 107). Implemented within an existing primary care HIV clinic located in the East Bay region of the greater San Francisco Bay Area, CRUSH aimed to serve patients receiving HIV care and treatment in the existing clinic and expand services to serve young people who were HIV-uninfected, but at risk for HIV infection. It provided sexual health services to a population of young people in a community where comprehensive HIV prevention and sexual health services for HIV-negative participants, including the provision of post- and pre-exposure prophylaxis (PEP and PrEP), were not yet readily available. Importantly, although our study was located in close geographic proximity to San Francisco, a site of HIV activism and sizable gay community, historically, the East Bay's public health infrastructure to support HIV prevention and care is far less resourced than the one in San Francisco, thus creating a significant need for sexual health programming offered through the CRUSH project. Most participants continued to use PrEP after they officially ended participation in the study. CRUSH study personnel provided insurance navigation services to facilitate on-going access to PrEP at no or low-cost.

### Procedures

The multi-method evaluation of the project included qualitative in-depth interviews conducted with CRUSH participants. We briefly outline the CRUSH project eligibility criteria here: ages 18 to 29, able to provide consent to participate in a research study in English or Spanish and receiving HIV care as a patient within the on-site youth HIV clinic (Downtown Youth Clinic) or self-identified as “at-risk” for HIV infection. For this analysis, we drew a sample of key informants from a larger pool of over 300 young people who enrolled in the CRUSH project. We purposively selected the key informants based on the recommendations of clinic staff who conducted an initial intake and study coordinators who conducted the baseline and follow-up assessments. Prospective key informants were selected because they were talkative, open, and comfortable with participation in research. Recommendations for key informants were vetted by first (KK) and second (XE) authors. Eligibility criteria were designed to be inclusive of participants at all levels of engagement in the CRUSH project. Although there were no specific racial/ethnic inclusion criteria, we oversampled African American and Latino participants and sought to capture the maximum variation with respect to PrEP utilization and other sexual health outcomes—such as repeated positive sexually transmitted infection (STI) test results during their participation in the CRUSH study.

Prior to initiating the interviews, individuals were allowed sufficient time to review the study information sheet and make an informed decision regarding their participation. We were granted permission to utilize verbal consent due to the privacy risks associated with the study. The first and second authors (KK and XE) conducted interviews jointly whenever possible, however the majority of the interviews was conducted by XE. XE is a Latino gay man who had over 5 years of experience providing HIV prevention, testing, and linkage services in the East Bay prior to joining the research team. He was trained in qualitative interview methods over the course of a year by the first author prior to starting the data collection described in this manuscript. He was encouraged to lead the interviews as he was a near-peer, having recently aged out of the eligibility range for this study and had multiple occasions to meet and build rapport with our research participants. KK is a white, heterosexual woman trained in anthropology and communication studies who has over 15 years of experience interviewing people affected by HIV. Interviews lasted between 60 and 90 min, took place in-person, in a private space, and were audio-recorded. The interview guide was designed to elicit narratives related to the following key areas of interest: learning about PrEP, motivations to take PrEP, concerns about PrEP, benefits of PrEP, negative or unforeseen impacts of PrEP, sexual experiences while on PrEP, sexual health services, including STI and HIV testing, and relationships. During the interview, spontaneous modifications were made when appropriate e.g., dropping questions that were not relevant to the participant. Following the interview, the participants were asked to complete a short demographic questionnaire. At the end, participants were given $40 in cash for their participation. All recruitment and study procedures were approved by the Institutional Review Boards at the University of California, San Francisco and Alta Bates Summit Medical Center in Oakland, the collaborating research site.

### Analysis

Audio-files were professionally transcribed verbatim, de-identified, and uploaded into Dedoose, a web-based data management program used to facilitate the organization and analysis of qualitative data (Version 7.5.6, 2017). We undertook an iterative analytic process beginning with the write-up of a fieldnote following each interview to outline the basic information conveyed in the interview as well as to capture early impressions, observations, and ideas that emerged. The more formal process of coding the transcripts began with reading aloud a subset of the transcripts to derive an initial set of codes which were then refined over time. Each interview was assigned a primary analyst and a secondary reviewer. This process facilitated a shared understanding of code application and when discrepancies emerged, we resolved them through discussion and consensus. For this analysis, we systematically reviewed the coded excerpts labeled *serodivide*, which we defined as narratives illustrating bridging the divide or increasing the divide, distilling ideas, observations, and propositions into tables. We used the tables to guide the identification of patterns across cases (108, 109). Finally, excerpts were selected to illustrate the main findings and to expand upon the selected themes.

Note, our interview guide included a general line of questioning about sexual practices following initiation of PrEP as well as a specific question to more directly elicit narratives about changes to partner selection based on serostatus. These questions were typically phrased as follows: “How has PrEP impacted your romantic or dating life?” and “How has being on PrEP changed your feelings or thoughts about dating a person who's positive, if at all?” Thus, partner selection narratives occurred in different parts of the interview, not just in response to our direct question. Of the 29 participants represented in this analysis, we applied the code “serodivide” in 24 individual cases. In over half of the coded excerpts, participants spontaneously talked about partner selection based on serostatus. The excerpts coded with “serodivide” were often co-occurring with “HIV attitudes” or “sex and dating narratives.”

To protect the identity and confidentiality of study participants, the names of our key informants have been replaced with pseudonym and their ages are represented as a range. We use the term *adolescent* for key informants ages 18 to 24 years old and *young adult* for key informants ages 25 to 29 years old.

## Findings

We conducted 37 in-depth interviews with 29 young G/MSM aged 18 to 29 years old between February, 2015 and January, 2016. We conducted repeat interviews with 8 participants which allowed us to explore changes over time (an average of 9 months between the first and second interview and we interviewed two informants three times over a 24-month period). All participants were HIV-uninfected, reported sex with other men and had initiated PrEP use. Demographic characteristics of the participants included in this analysis can be found in Table 1.

**Table 1:** Description of the study sample (*N* = 29).

**G/MSM Cohort demographics**	**Overall**	
	***N***	**(%)**
**AGE**		
18-24	11	37.93
25-29	18	62.07
**GENDER**		
Male	29	100.00
**RACE/ETHNICITY**		
Black	8	27.58
Latino	11	37.93
White	6	20.69
Asian and Pacific Islander	2	6.90
Two or more races	2	6.90
**SEXUAL IDENTITY**		
Bisexual	3	10.35
Gay (includes queer)	25	86.20
Other MSM	1	3.45
**EDUCATION**		
High school diploma	3	10.35
Some college, less than 4Yr degree	16	55.17
Bachelor's degree	7	24.13
Graduate school	3	10.35

During our analysis, we identified trends shifting away from serosorting toward its opposite, something we labeled “seromixing.” We defined seromixing as a practice of electing to have partners with a different serostatus. One participant spoke of “losing the phobia” (of having sex with a person living with HIV) when referring to his shift from serosorting to seromixing. We used his narrative describing a trajectory toward becoming less phobic as our blueprint for examining and classifying all other narratives on serosorting/seromixing practices. Below we describe whether, how, and why the use of PrEP influenced conceptualizations of HIV-risk, serosorting, and HIV-related stigma. We present three orientations to the serodivide concept: (1) *maintaining the phobia* includes justifications for the continued vigilance and exclusion of people living with HIV as viable sex or romantic partners, (2) *loosening/relaxation of phobia* among men accounts of deeper reflection about their stance on serosorting and its implications for future sexual and/or romantic partnerships, and (3) *losing the phobia* accounts of men letting go of serosorting practices that reinforced HIV-related stigma in the context of PrEP use.

Most men noted decreased importance of serostatus in selecting romantic and sexual partners, and in some cases, profoundly changed attitudes with regards to differentiating between bodies with and without HIV infection. These overarching themes offer explanatory accounts associated with the phenomenon of PrEP use and its contribution to bridging or maintaining the HIV serodivide. Overall, our data indicate a trend toward seromixing. The majority of participants spoke of changing or changed attitudes about intentionally accepting rather than rejecting a person living with HIV as a sex partner.

### Maintaining the phobia

A handful of participants (*n* = 4) articulated an unchanged discomfort with the idea of seromixing. Notably, no one in this category used the term “phobia” or identified as “phobic” during discussions about the idea of having sex with people living with HIV. The language and expressions used by those who continued to serosort while on PrEP included feeling “scared” or being “turned off” by those perceived to be “not clean”—a euphemism used when referring to someone with a sexually transmitted infection and/or a person living with HIV. Participants spoke of HIV in a way that construed it as an on-going threat that continued to influence how they made decisions about sexual partners. For these men, the belief in PrEP's ability to prevent HIV was not powerful enough to inoculate against existing fears and anxieties about contracting HIV. They expressed concerns about these consequences e.g., “I don't know if it's worth the risk” or “what if…” Accounts like these illustrate some men's desire to hold tightly to current practices and perspectives on serosorting—what we call a “hell no” stance toward seromixing as articulated by Octavio:

I met a guy before that was positive, undetectable as well. But, like, I didn't even kiss him or anything. Even this guy I just met. Like, with him, I was like, no, like, hell no. I'm not doing anything with you. (Octavio, Young Adult, Latino, Gay Male)

Jorge explained his position that he would not intentionally have sex with a person living with HIV by using the euphemism “why poke the bear?”

I guess, going in knowing—I'm not sure if that's something I would ever do, even though I have the, you know, protection there. Why poke the bear? (Jorge, Young Adult, Latino, Gay Male)

It is possible Jorge perceived himself as behaving responsibly, rather than seeing himself as phobic, because of his stance on exclusive serosorting. For those who considered prospective sex partners as dangerous, it is perhaps unsurprising and/or understandable that they would chose to both prevent HIV with PrEP and to continue to actively serosort. In the case of Pablo, we noted more introspection about the consequence of his decision to exclude PLWH. Below he acknowledged that he felt uneasy or conflicted e.g., “it may be sucks” about his decision to exclusively hook up with people who are “clean.”

I feel like it maybe sucks but if I'm going to hook up with someone it's just going to be someone who's clean. (Pablo, Young Adult, Latino, Gay Male)

### Loosening/relaxation of the phobia

The second category “loosening the phobia” consisted of narratives elicited from participants (*n* = 6) who, in response to our question about how PrEP changed their thoughts or feelings about dating a person living with HIV, provided answers indicating they had not given the idea any prior thought and were thus producing a reaction with us present. These reactions were patterned around the notion that they were “warming up” to the idea of seromixing. These participants expressed a type of contingent acceptance of PLWH as prospective casual sex partners, lovers, and/or viable romantic partners. Men contemplated when to be more inclusive when selecting romantic and/or sex partners. In these interviews, men expressed less outright resistance to the idea of seromixing than those who were maintaining the phobia. For example, these men uniformly described carving out exceptions for partnering with people living with HIV. Mario, a young adult and Latino gay male, exemplified this perspective:

Mario: I think that if I really love that person, I would [consider dating somebody who was positive]. I believe that I would.

Interviewer: Do you think that's changed because of PrEP? Or, have you always felt that way?

Mario: Yeah, definitely. I mean, no, definitely not. Just the fact that there is something like PrEP. Okay, so, we don't always have to worry about condoms or, like, blood, transferring blood, or anything like that. So, we don't always have to worry about that. Maybe that could be a big thing. I mean, I don't know the whole logistics of always being on PrEP, like, 24/7 for the rest of your life. I don't know about all of that. But, I'm just saying, like, okay, maybe we could at least get to know each other first. If it comes down to that, we'll make it work. If you love each other, you can make it work. (Mario, Young Adult, Latino, Gay Male)

We noted that some participants formulated their responses to partner selection questions by initially making a third-person, generalized observation before shifting to first person statements. Carlos provided an example of this type of response when he said, “…people now are more aware…” and then goes on to classify his own experience as being more aware implying that his behavior and decision-making processes are part of a larger trend:

I think that people now are more aware… Actually, even a couple of years ago, it was strange for me because, I don't know anyone who - I mean, I've met people who are HIV positive. But, like, I've never actually, been very close to someone who is HIV positive. And I've had friends who have gone on dates and then find out that the person is HIV positive and they're just kind of like, “Oh, no. I don't want that.” And I think that being on PrEP has sort of changed my perspective. And I feel that, if anything, someone who is able to disclose their HIV status, they're probably more safe than someone who thinks they're HIV-negative. (Carlos, Young Adult, Latino, Gay Male)

Carlos explained further that he believed that someone who was able to disclose their HIV status has both courage and are were likely to take care of themselves. These attributes merited his respect and helped to shift his simplistic perceptions about the HIV disease. In his case, becoming more informed about HIV and initiating PrEP use loosened his stance on the “dangers” of seromixing.

In the illustrative case below, our line of questioning about how serosorting fits into the respondent's life while on PrEP created an opportunity for him (and others) to work out and articulate a position on the subject. It was not until the question was posed and upon reflection that Paul ultimately concluded that he “would feel safer” having sex with a person living with HIV now that he was on PrEP. Like other participants we classified in this category, Paul had not changed his partner selection practices in any obvious ways as a result of PrEP use. When pressed to reflect, men in this category offered a softened stance on seromixing, albeit one that was theoretical.

I don't think I would casually go have sex with someone that was HIV-positive, to be honest with you… But, I mean, yeah, I guess if I was dating someone and, like, I really felt strongly about my feelings for them, being [on] PrEP would definitely make me feel easier about having sex with them. I probably wouldn't have sex with them if I didn't have something like PrEP. … So, in a way, yeah, I would actually - now that I've, like, thought through it, I think it would make me feel easier about having sex with them. (Paul, Adolescent, Multi-Racial, Gay Male)

Gabriel also believed that more people living with HIV were “putting themselves out there.” With more willingness to seromix among PrEP users, it may be that PLWH are more willing to be open about their own serostatus, which in turn, may be evidence of a collapsing serodivide.

Interviewer: How has your experience on PrEP changed your feelings, thoughts, or concerns about dating a person who's positive?

Gabriel: I think I'm open to it. And I think in the past I would say that I'm open to it, but when it came down to it, I would be, like, a little bit nervous and unsure. …I think I've noticed that on these sort of meeting sites I've been seeing more and more pos people that are sort of [unabashedly], you know, putting themselves out there. And, so, I don't know if that's, like, a reflection of just the times and society in the Bay, or maybe it does have a good amount to do with PrEP because it is—it is a big aspect of the Bay Area right now. But, yeah, I think it goes both ways. I think people that are pos are less afraid to seek out partners or encounters from either other pos people or non, and then people that are non-pos are open to sort of seeing where things go with people that are pos. (Gabriel, Young Adult, Latino, Gay Male)

### Losing the phobia

We classified the majority of participants (*n* = 13) into the “losing the phobia” category. Participants in this category described previously rejecting people living with HIV as sex partners, explaining that they were not comfortable partnering with a person known to be HIV-infected. In the words of one participant, learning about a potential partner's HIV-positive status had been a “deal breaker” prior to using PrEP. We were struck by the dramatic shift away from this attitude in many accounts. For example, Jose, an adolescent Latino gay man offered an account to explain his conceptualization of HIV, HIV risk, and people living with HIV before and after using PrEP:

Interviewer: Do you think PrEP has changed your feelings, thoughts or beliefs about dating someone who is positive?

Jose: Very much—because I feel that I was in such a dark place in my life… I wanted to avoid anything or anyone with HIV. And, I was very, very aggressive about it. If I found out someone was HIV positive, I would completely stop talking to the person. … And it made me feel very, very bad—that I shouldn't judge a person because of the disease and I shouldn't point fingers, or I shouldn't point out that oh maybe you should wear a condom next time. I felt terrible.…It was almost like someone just completely blinded me.…I don't know, it probably sounds very self-centered, but it made me feel good about myself that I could [now] see people for who they are, not for what they suffer from or what they're going through, and it made me feel very good about myself. (Jose, Adolescent, Latino, Gay Male)

In the case of Roberto, an adolescent Latino gay male, the participant who articulated the notion of “losing the phobia,” he explained to the interviewer that PrEP influenced his decisions about who to hook up with. His account was straightforward in that he acknowledged his prior “phobia” and couched it as problematic:

I did have a phobia of people who were positive, and I remember even on the hookup apps people would say they were positive, and I remember times saying like I would hate to come off this way, but I just don't feel comfortable with that. So, and I've had three conscious experiences with someone who told me that they were positive, undetectable, and we had sex. I mean, I made that conscious decision. So, that changed, and the way I see people who are positive has changed.…I'm much less pos-phobic. I'm like tell me if you're positive. That's fine. We can still hook up. Let's just use a condom. Or I'm on PrEP. That's fine. … So, my communications are different. But I'm like if someone tells me and they're open, …I'm like, OK, cool. Well, I trust you because you're honest. You're not trying to deceive me. So, I'm down. (Roberto, Adolescent, Latino, Gay Male)

The above account illustrates Roberto's ambivalence about having anxieties about having sex with a person living with HIV. The psychosocial concept of a phobia, as mentioned above, helped us to frame the orientation we applied while examining serosorting narratives.

In another case, Thomas provided a clear example of how PrEP use helped to de-emphasize the centrality of HIV serostatus when sex was being negotiated. In the account below, Thomas, an adolescent Black gay male, indicated that with “the whole negative-positive thing, everybody's just neutral with it [now].” This participant observed a shift in how people related to information about one's HIV serostatus. As mentioned earlier, he too initially referred to “everybody”—the safety of the third person—“everybody's neutral” and “there's a lack of concern” before he then personalized the account: “I told my sex partner I was on PrEP.” His disclosure as a PrEP user may have facilitated his partner's disclosure of his seropositive HIV status. He expressed feeling pleased that he and his partner could “see each other as people as opposed to our HIV status.” He further pointed out that this was working in both directions: a partner who was HIV-infected was relieved to know that Thomas was on PrEP: “For both of us, PrEP made it super-easy to see each other as people as opposed to our HIV status.”

Interviewer: How has PrEP affected your sexual life?

Thomas: For relationships, [now?] I guess the whole negative-positive thing, everybody's just neutral with it. So, there's definitely a lack of concern of whether or not they're HIV-positive…There was one relationship where it was like I told them I was on PrEP, and they felt super-relieved because they hadn't told me that they were HIV-positive. Because of that, I want to say it's sort of, for both of us, it made it super-easy for us to see each other as people as opposed to our HIV status. I think—I can't really say, but what I got from him was like it was—there's always this concern about how he was going to tell somebody about his status and stuff. And it was pleasant to just see a functional dating process without having to go through all the nitty-gritty details about sexual health. (Thomas, Adolescent, Black, Gay Male)

Finally, we offer an excerpt from a repeat interview with Roberto reflecting on how PrEP has changed the way he conceptualizes HIV and his relationship to people living with it. He responded to the question, “What does HIV mean to you now?” by stating:

Yeah, stigma has gone away overall. PrEP has reduced my fear of becoming positive but it's also changed my conversation and it's changed my outlook on how I see people who are living with HIV. It's been overall a more positive shift in how I view it and I just think I have a healthier outlook on how I can keep myself negative, but also how I respond to someone who is positive. (Roberto, Adolescent, Latino, Gay Male)

## Discussion

“*Sexual cultures are by no means unchanging*” (98).

The medicalization of HIV prevention has created an opening for some men to relate to HIV differently (96). In this article, we focused on how men related to prospective sexual and/or romantic partners who were living with HIV. We noted cases where men shifted from choosing to exclude, to actively choosing to include these types of partners. Participants attributed their change of attitude to their use of PrEP. Decreased anxiety about contracting HIV translated into opening up discussions and acting on opportunities to “hook up” with a PLWH. In these situations, PrEP use prompted an examination about the way they had previously made sense of HIV, and their previous justifications for excluding certain people as viable partners. These shifts sometimes included raising one's awareness about the current state of HIV treatment and the contemporary life of a person living with HIV—that PLWH can live a full lifespan, that HIV may be a chronic illness, and that effective, tolerable HIV treatments exist. Those in the “losing the phobia” category came to have a different way of assessing the possibility of HIV acquisition. With this new reality, they reflected on their prior ways of categorizing and excluding—some with embarrassment, and others with more self-compassion and ability to justify why they would not be able to “go through with it.”

The “losing the phobia” perspective gained momentum in part due to a privileging of both the scientific knowledge about the efficacy of PrEP as well as a belief that public discourse was changing whereby PLWH could be more open about their serostatus. This openness about living with HIV and acceptance of those PLWH as viable partners appeared to us to have a de-stigmatizing effect among the men in our study. For example, Jose and others like him, provided accounts that indicated they no longer faced the “micro-dilemma” (9) of whether to accept or reject a person based on their HIV serostatus.

As with the decision to serosort, the decision to seromix was done with intentionality. Participants who moved from serosorting to seromixing did so after considering why they enacted their prior behaviors and asked themselves whether that practice currently served them. However, the factors considered when calculating risk were different among various participants. For those who maintained strict serosorting practices, we shed light on understanding why they continued to do so. These participants' understandings of HIV risk were not erased under the promise of PrEP and perhaps after years of avoiding people living with HIV as sex partners, notions about infectiousness had become habituated for these men and appeared to more difficult to shift.

Our study has limitations. We spoke to young G/MSM in a particular time and place with its own particular social rules and norms about sex, dating, and partnerships. These contextual factors influence social norms which may be similar or different in other urban environments with a sizable gay community in the process of embracing widespread use of PrEP. Ordinarily, the small sample size upon which qualitative findings are derived is often described as a limitation with regard to generalizability. We wish to emphasize, however, that while we do not claim that our findings are generalizable to all other young G/MSM who use PrEP, we do imagine that the subtle to significant shift in attitudes about seromixing may likely shared by other G/MSM PrEP users in the United States and elsewhere. We encourage further research on this phenomenon to test this assumption. An additional area for future research would be to include the perspective of young G/MSM living with HIV to understand whether and how widespread PrEP use has impacted their dating and sexual experiences. For example, to what extent does the continuum we described above of maintaining to losing the “phobia” of partnering with persons of the opposite status resonate for men who are living with HIV?

Understanding how young gay and bisexual men make sense of HIV in the context of PrEP has implications for program implementation, outreach, and community education efforts. For example, one such implication includes the possibility that any reduction in HIV-related stigma resulting from greater PrEP use and seromixing behaviors may also lead to greater numbers of persons overcoming stigma-related barriers to test and, perhaps, to test more frequently for HIV. On the other hand, public health experts may interpret a trend away from serosorting and toward seromixing as cause for concern, particularly in cases of sub-optimal PrEP adherence.

Emergent trepidations following the introduction of new technologies and interventions to address *risky behaviors* is the norm. PrEP is not an exception. Concerns have been raised about the impact of PrEP on risk perception and risk behaviors. To date, however, data do not support a significant increase in risk for HIV among people who use PrEP as directed although there are documented cases of HIV infection among PrEP users with high levels of adherence (110–112). Holt et al takes this idea further in a recent publication in *The Lancet* describing a phenomenon termed “community-level risk compensation” whereby they observed between 2013 and 2017 a concurrent decline in condom use among HIV-negative gay men *not using* PrEP accompanying rapid increases in PrEP use in two major cities in Australia (113). Qualitative research to contextualize the decline in condom use among gay men in urban areas in Australia and to ascertain the extent that this observation is part of a general downward trend in condom use and/or is in direct response to the promise of biomedical HIV prevention strategies such as PrEP and TasP is needed. The behavioral surveillance data collected by Holt et al do not necessarily indicate an increase in seromixing or an equivalent decentralization of HIV serostatus in partner selection. If and when behavioral surveillance data evidence a trend toward increases in seromixing, we would ask researchers and public health officials to balance the concerns with the social benefits of normative seromixing.

The exclusion of people living with HIV as viable sexual partners has led to unintended consequences such as fear of rejection by sexual partners, poor self-image and loss of libido (23, 25). These disadvantages of serosorting have heretofore been under-acknowledged in the literature, which has focused almost exclusively on ascertaining the *biological* risks and benefits of serosorting. PrEP use, in the cases described above, contributes to diminishing fears of contracting HIV and in many cases, a de-emphasis in sexual exchanges of HIV serostatus. For these reasons, significant social benefits of widespread PrEP use may include greater acceptance by HIV-uninfected people of PLWH as viable sexual and romantic partners and, ultimately, reduced HIV-related stigma. The disruption to serosorting practices as a result of HIV pre-exposure prophylaxis may be a welcome opportunity to create a new set of social and sexual relationships for those living with and affected by HIV.

## Ethics statement

This study was carried out in accordance with the ethical guidelines and regulations for research involving human subjects provided by the National Institute of Health. The protocol was approved by both the Institutional Review Boards at the University of California, San Francisco and Alta Bates Summit Medical Center in Oakland, the collaborating research site. All subjects gave written informed consent in accordance with the Declaration of Helsinki.

## Author contributions

KK contributed substantially to the conception and design of the study, the acquisition of data, the analysis, and interpretation, drafting the manuscript and provided final approval of the version to publish. XE contributed substantially to the acquisition of the data, the analysis, and interpretation, as well as drafting the manuscript. M-SK contributed substantially to the conception and design of the study, and provided critical revision of the manuscript. IU contributed substantially to the conception and design of the study, and well as served as the project manager for the larger parent study of which this analysis is one part. JB, RG, and JM were the principal investigators for the parent study, oversaw the research process and ensured consistent, ethical, and rigorous study procedures were followed. All authors contributed to manuscript revision, read, and approved the submitted version.

### Conflict of interest statement

The authors declare that the research was conducted in the absence of any commercial or financial relationships that could be construed as a potential conflict of interest.

## References

[1] Race K Click here for HIV status: Shifting templates of sexual negotiation. Emot Space Soc. (2010) 3:7–14. 10.1016/J.EMOSPA.2010.01.003

[2] Kippax S Race K. Sustaining safe practice: twenty years on. Soc Sci Med. (2003) 57:1–12. 10.1016/S0277-9536(02)00303-9 12753812

[3] Halperin D What Do Gay Men Want? Ann Arbor, MI: University of Michigan Press (2007).

[4] Parsons JT Vicioso K Brief Encounters: The Roles of Public and Commercial Sex Environments in the Sexual Lives of HIV-Positive Gay and Bisexual Men. In: HIV+ Sex: The Psychological and Interpersonal Dynamics of HIV-Seropositive Gay and Bisexual Men's Relationships. Washington, DC: American Psychological Association (2005). p. 183–200.

[5] Parsons JT Schrimshaw EW Wolitski RJ Halkitis PN Purcell DW Hoff CC. Sexual harm reduction practices of HIV-seropositive gay and bisexual men: serosorting, strategic positioning, and withdrawal before ejaculation. AIDS (2005) 19 (Suppl. 1):S13–25. 10.1097/01.aids.0000167348.15750.9a 15838191

[6] Vallabhaneni S Li X Vittinghoff E Donnell D Pilcher CD Buchbinder SP. Seroadaptive practices: association with HIV acquisition among HIV-negative men who have sex with men. PLoS ONE (2012) 7:e45718. 10.1371/journal.pone.0045718 23056215 PMC3463589

[7] Cairns G When Serosorting is Seroguessing. Nam Aidsmap (2007). Available online at: http://www.aidsmap.com/When-serosorting-is-seroguessing/page/1427776

[8] Zablotska IB Imrie J Prestage G Crawford J Rawstorne P Grulich A. Gay men's current practice of HIV seroconcordant unprotected anal intercourse: serosorting or seroguessing? AIDS Care (2009) 21:501–10. 10.1080/09540120802270292 19266409

[9] Fontdevila J Framing dilemmas during sex: a micro-sociological approach to HIV risk. Soc Theory Health (2009) 7:241–63. 10.1057/sth.2009.2

[10] Ciccarone DH Kanouse DE Collins RL Miu A Chen JL Morton SC. Sex without disclosure of positive HIV serostatus in a US probability sample of persons receiving medical care for HIV infection. Am J Public Health (2003) 93:949–54. 10.2105/AJPH.93.6.949 12773361 PMC1447876

[11] Cox J Beauchemin J Allard R. HIV status of sexual partners is more important than antiretroviral treatment related perceptions for risk taking by HIV positive MSM in Montreal, Canada. Sex Trans Infect. (2004) 80:518–23. 10.1136/sti.2004.011288 15572627 PMC1744941

[12] McConnell JJ Bragg L Shiboski S Grant RM. Sexual seroadaptation: lessons for prevention and sex research from a cohort of HIV-positive men who have sex with men. PLoS ONE (2010) 5:e8831. 10.1371/journal.pone.0008831 20098616 PMC2809110

[13] Philips SS Yu X Donnell D Vittinghoff E Buchbinder S Serosorting is associated with a decreased risk of HIV seroconversion in the EXPLORE study cohort. PLoS ONE (2010) 5:e12662 10.1371/journal.pone.0012662 20844744 PMC2936578

[14] Hamel L Firth J Hoff T Kates J Levine S Dawson L HIV/AIDS in the Lives of Gay and Bisexual Men in the United States|The Henry J. Kaiser Family Foundation (2014). Available online at: https://www.kff.org/hivaids/report/hivaids-in-the-lives-of-gay-and-bisexual-men-in-the-united-states/

[15] Frye V Wilton L Hirshfield S Chiasson MA Lucy D Usher D. Preferences for HIV test characteristics among young, Black Men Who Have Sex With Men (MSM) and transgender women: Implications for consistent HIV testing. PLOS ONE (2018). 13, e0192936. 10.1371/journal.pone.0192936 29462156 PMC5819791

[16] Sheon N Crosby GM. Ambivalent tales of HIV disclosure in San Francisco. Soc Sci Med. (2004) 58:2105–18. 10.1016/j.socscimed.2003.08.026 15047070

[17] Courtenay-Quirk C Wolitski RJ Parsons JT Gómez CA. Is HIV/AIDS stigma dividing the gay community? Perceptions of HIV-positive men who have sex with men. AIDS Educ Prev. (2006) 18:56–67. 10.1521/aeap.2006.18.1.56 16539576

[18] Murphy DA De Wit JBF Donohoe S Adam PCG. The need to know: HIV status disclosure expectations and practices among non-HIV-positive gay and bisexual men in Australia. AIDS Care (2015) 27:90–8. 10.1080/09540121.2015.1062077 26616130 PMC4685622

[19] Persson A Ellard J Newman CE Bridging the HIV divide: stigma, stories and serodiscordant sexuality in the biomedical age. Sex Cult. (2016) 20:197–213. 10.1007/s12119-015-9316-z

[20] Sengupta S Strauss RP Miles MS Roman-Isler M Banks B Corbie-Smith G. A conceptual model exploring the relationship between HIV stigma and implementing HIV clinical trials in rural communities of North Carolina. N C Med J. (2010) 71:113–22. Available online at: http://www.pubmedcentral.nih.gov/articlerender.fcgi?artid=3037544&tool=pmcentrez&rendertype=abstract 20552760 PMC3037544

[21] Koester KA Erguera XA Myers JJ “PrEP Makes My Relationship Even More Normal:” the discursive production of hope in the context of HIV pre-exposure prophylaxis among young adults with partners living with HIV infection. In: Persson A Hughes SD, editors. Cross-Cultural Prespectives on Couples with Mixed HIV Status: Beyond Positive/Negative Vol. 2. Switzerland: Springer International Publishing (2017). p. 23–35. 10.1007/978-3-319-42725-6

[22] Frost DM Stirratt MJ Ouellette SC. Understanding why gay men seek HIV-seroconcordant partners: Intimacy and risk reduction motivations. Cult Health Sex. (2008) 10:513–27. 10.1080/13691050801905631 18568873

[23] Bourne A Hickson F Keogh P Reid D Weatherburn P. Problems with sex among gay and bisexual men with diagnosed HIV in the United Kingdom. BMC Public Health (2012) 12:916. 10.1186/1471-2458-12-916 23107161 PMC3503855

[24] Skinta MD Brandrett BD Schenk WC Wells G Dilley JW. Shame, self-acceptance and disclosure in the lives of gay men living with HIV: an interpretative phenomenological analysis approach. Psychol Health (2014) 29:583–97. 10.1080/08870446.2013.871283 24303867

[25] Rohleder P McDermott DT Cook R. Experience of sexual self-esteem among men living with HIV. J Health Psychol. (2017) 22:176–85. 10.1177/1359105315597053 26238342

[26] Watts C Zimmerman C Eckhaus T Nyblade L Modelling the Impact of Stigma on HIV and AIDS Programmes: Preliminary Projections for Mother-to-Child Transmission (2010). Available online at: https://www.icrw.org/wp-content/uploads/2016/10/Modelling-the-Impact-of-Stigma-on-HIV-and-AIDS-Programmes.pdf

[27] de Santis JP Florom-Smith A Vermeesch A Barroso S DeLeon DA Motivation, management, and mastery: a theory of resilience in the context of HIV infection. J Am Psychiat Nurses Assoc. (2013) 19:36–46. 10.1177/1078390312474096 PMC3773721 23392433

[28] Harris GE Larsen D. Understanding hope in the Face of an HIV diagnosis and high-risk behaviors. J Health Psychol. (2008) 13:401–15. 10.1177/1359105307088143 18420773

[29] Smit PJ Brady M Carter M Fernandes R Lamore L Meulbroek M. HIV-related stigma within communities of gay men: a literature review. AIDS Care (2012) 24:405–12. 10.1080/09540121.2011.613910 22117138 PMC3379736

[30] Hood JE Buskin SE Dombrowski JC Kern DA Barash EA Katz DA Dramatic increase in preexposure prophylaxis use among MSM in King County, Washington. AIDS (2015) 30:515–9. 10.1097/QAD.0000000000000937 26562845

[31] Parsons JT Rendina HJ Lassiter JM Whitfield THF Starks TJ Grov C. Uptake of HIV Pre-Exposure Prophylaxis (PrEP) in a national cohort of gay and bisexual men in the United States. JAIDS (2017) 74:285–92. 10.1097/QAI.0000000000001251 28187084 PMC5315535

[32] Van De Ven P Prestage G Crawford J Grulich A Kippax S Sexual risk behaviour increases and is associated with HIV optimism among HIV-negative and HIV-positive gay men in Sydney over the 4 year period to February 2000. AIDS (2000) 14:2951–3. 10.1097/00002030-200012220-00023 11153682

[33] Bird JDP Morris JA Koester KA Pollack LM Binson D Woods WJ “Knowing Your Status and Knowing Your Partner's Status Is Really Where It Starts”: a qualitative exploration of the process by which a sexual partner's hiv status can influence sexual decision making. J Sex Res. (2017) 54:784–94. 10.1080/00224499.2016.1202179 27485155 PMC5290286

[34] Blythe SP Castillo-Chavez C. Like- with- like preference and sexual mixing models. Math Biosc. (1989) 96, 221–38. 2520199 10.1016/0025-5564(89)90060-6

[35] Dawson JM Fitzpatrick RM Reeves G Boulton M McLean J Hart GJ. Awareness of sexual partners' HIV status as an influence upon high-risk sexual behaviour among gay men. AIDS (1994) 8:837–41. 10.1097/00002030-199406000-00018 8086144

[36] Boily MC Lowndes CM Gregson S Population-level risk factors for HIV transmission and “the 4 cities study”: temporal dynamics and the significance of sexual mixing patterns [5]. AIDS (2002) 16:2101–2. 10.1097/00002030-200210180-00025 12370519

[37] Butler DM Smith DM. Serosorting can potentially increase HIV transmissions. AIDS (2007) 21:1218–20. 10.1097/QAD.0b013e32814db7bf 17502737

[38] Wilson DP Regan DG Heymer KJ Jin F Prestage GP Grulich AE. Serosorting may increase the risk of HIV acquisition among men who have sex with men. Sex Transm Dis (2010) 37:13–7. 10.1097/OLQ.0b013e3181b35549 20118674

[39] Kennedy CE Bernard LJ Muessig KE Konda KA Akl EA Lo YR. Serosorting and HIV/STI infection among HIV-negative MSM and transgender people: a systematic review and meta-analysis to inform WHO guidelines. J Sex Trans Dis. (2013) 2013:1–8. 10.1155/2013/583627 26316960 PMC4437432

[40] Anderson RM Medley GF May RM Johnson AM. A preliminary study of the transmission dynamics of the human immunodeficiency virus (HIV), the causative agent of AIDS. IMA J Math Appl Med Biol. (1986) 3:229–63. 3453839 10.1093/imammb/3.4.229

[41] Anderson RM May RM. Epidemiological parameters of Hiv transmission. Nature (1988) 333:514–9. 10.1038/333514a0 3374601

[42] Anderson RM Ng TW Boily MC May RM. The influence of different sexual-contact patterns between age classes on the predicted demographic impact of AIDS in Developing Countries. Ann N Y Acad Sci. (1989) 569:240–74. 10.1111/j.1749-6632.1989.tb27374.x 2698092

[43] Laumann EO Gagnon JH Michaels S Michael RT Coleman JS. Monitoring the AIDS Epidemic in the United States: a Network Approach. Science (1989) 244:1186–9. 10.1126/science.2543079 2543079

[44] Gupta S Anderson RM May RM. Networks of sexual contacts. AIDS (1989) 3:807–18. 10.1097/00002030-198912000-00005 2517202

[45] Anderson R Gupta S Ng W. The significance of sexual partner contact networks for the transmission dynamics of HIV. J Acq Immune Def Syndr. (1990) 3:417–29. 2179528

[46] Lemp GF Porco TC Hirozawa AM Lingo M Woelffer G Hsu LC. Projected incidence of AIDS in San Francisco: the peak and decline of the epidemic. J Acq Immune Def Syndr Hum Retrovirol. (1997) 16:182–9. 10.1097/00042560-199711010-00007 9390570

[47] Ekstrand ML Stall RD Paul JP Osmond DH Coates TJ Gay men report high rates of unprotected anal sex with partners of unknown or discordant HIV status. AIDS (1999) 13:1525–33. 10.1097/00002030-199908200-00013 10465077

[48] Catania JA Osmond D Stall RD Pollack L Paul JP Blower S. The continuing HIV epidemic among men who have sex with men. Am J Public Health (2001) 91:907–14. 10.2105/ajph.91.6.907 11392933 PMC1446467

[49] Schwarcz S Kellogg T McFarland W Louie B Kohn R Busch M. Differences in the temporal trends of HIV seroincidence and seroprevalence among sexually transmitted disease clinic patients, 1989-1998: application of the serologic testing algorithm for recent HIV seroconversion. Am J Epidemiol. (2001) 153:925–34. 10.1093/aje/153.10.925 11384946

[50] Chen SY Gibson S Katz MH Klausner JD Dilley JW Schwarcz SK. Continuing increases in sexual risk behavior and sexually transmitted diseases among men who have sex with men: San Francisco, Calif, 1999-2001. Am J Public Health (2002) 92:1387–8. 10.2105/ajph.92.9.1387-a 12197957 PMC1447248

[51] Katz MH Schwarcz SK Kellogg TA Klausner JD Dilley JW Gibson S Impact of highly active antiretroviral treatment on HIV seroincidence among men who have sex with men: San Francisco. Am J Public Health (2002) 9292:388–94. 10.2105/ajph.92.3.388 PMC1447086 11867317

[52] Dukers NH Goudsmit J de Wit JB Prins M Weverling GJ Coutinho R. Sexual risk behaviour relates to the virological and immunological improvements during highly active antiretroviral therapy in HIV-1 infection. AIDS (2001) 15:369–78. 10.1097/00002030-200102160-00010 11273217

[53] Elford J Bolding G Sherr L High-risk sexual behaviour increases among London gay men between 1998 and 2001: what is the role of HIV optimism? AIDS (2002) 16:1537–44. 10.1097/00002030-200207260-00011 12131192

[54] Stolte IG Dukers NHTM Geskus RB Coutinho RA De Wit JBF. Homosexual men change to risky sex when perceiving less threat of HIV/*AIDS* since availability of highly active antiretroviral therapy: A longitudinal study. AIDS (2004) 18:303–9. 10.1097/00002030-200401230-00021 15075549

[55] Van de Ven P Rawstorne P Nakamura T Crawford J Kippax S HIV treatments optimism is associated with unprotected anal intercourse with regular and with casual partners among Australian gay and homosexually active men. Int J STD AIDS (2002) 13:181–3. 10.1258/0956462021924884 11860696

[56] Ostrow DE Fox KJ Chmiel JS Silvestre A Visscher BR Vanable P. Attitudes towards highly active antiretroviral therapy are associated with sexual risk taking among HIV-infected and uninfected homosexual men. AIDS (2002) 16:775–80. 10.1097/00002030-200203290-00013 11964534

[57] Elford J Hart G. If HIV prevention works, why are rates of high-risk sexual behavior increasing among MSM? AIDS Educ Prevent (2003) 15:294–308. 10.1521/aeap.15.5.294.23825 14516015

[58] Stockman JK Schwarcz SK Butler LM De Jong B Chen SY Delgado V HIV prevention fatigue among high-risk populations in San Francisco [4]. J Acquir Immune Defic Syndromes (2004) 35:432–4. 10.1097/00126334-200404010-00016 15097163

[59] Adam BD Husbands W Murray J Maxwell J. AIDS optimism, condom fatigue, or self-esteem? Explaining unsafe sex among gay and bisexual men. J Sex Res. (2005) 42:238–48. 10.1080/00224490509552278 19817037

[60] Elford J. Changing patterns of sexual behaviour in the era of highly active antiretroviral therapy. Curr Opin Infect Dis. (2006) 19:26–32. 10.1097/01.qco.0000199018.50451.e1 16374214

[61] Rowniak S. Safe sex fatigue, treatment optimism, and serosorting: new challenges to HIV prevention among men who have sex with men. J Assoc Nurses AIDS Care (2009) 20:31–8. 10.1016/j.jana.2008.09.006 19118769

[62] Shefer T Strebel A Jacobs J. AIDS fatigue and university students' talk about HIV risk. Afr J AIDS Res. (2012) 11:113–21. 10.2989/16085906.2012.698078 25859914

[63] Winkelstein W Jr Lyman DM Padian N Grant R Samuel M Wiley JA Sexual practices and risk of infection by the human immunodeficiency virus. The San Francisco Men's Health Study. JAMA (1987) 257:321–5. 10.1001/jama.257.3.321 3540327

[64] Winkelstein W Samuel M Padian NS Wiley JA Lang W Anderson RE. The San Francisco Men's Health Study: III. Reduction in human immunodeficiency virus transmission among homosexual/bisexual men, 1982-86. Am J Public Health (1987) 77:685–9. 10.2105/AJPH.77.6.685 3646848 PMC1647059

[65] Winkelstein W Wiley JA Padian NS Samuel M Shiboski S Ascher MS. The San Francisco Men's Health Study: continued decline in HIV seroconversion rates among homosexual/bisexual men. Am J Public Health (1988) 78:1472–4. 10.2105/AJPH.78.11.1472 3177723 PMC1350242

[66] Stall RD Hays RB Waldo CR Ekstrand M McFarland W The gay'90s: a review of research in the 1990s on sexual behavior and HIV risk among men who have sex with men. AIDS (2000) 14:S101–S114. 11086853

[67] Prestage G. Increase in unprotected anal intercourse with casual partners among Sydney gay men in 1996-98. Aust N Z J Public Health (1998) 22:814–8. 10.1111/j.1467-842X.1998.tb01499.x 9889449

[68] Dodds JP Mercey DE Parry JV Johnson AM. Increasing risk behaviour and high levels of undiagnosed HIV infection in a community sample of homosexual men. Sex Trans Infect (2004) 80:236–40. 10.1136/sti.2003.007286 15170012 PMC1744829

[69] Van De Ven P Kippax S Crawford J Rawstorne P Prestage G Grulich A In a minority of gay men, sexual risk practice indicates strategic positioning for perceived risk reduction rather than unbridled sex. AIDS Care (2002) 14:471–80. 10.1080/09540120220133008 12204150

[70] Mao L Crawford JM Hospers HJ Prestage GP Grulich AE Kaldor JM. “Serosorting” in casual anal sex of HIV-negative gay men is noteworthy and is increasing in Sydney, Australia. AIDS (2006) 20:1204–6. 10.1097/01.aids.0000226964.17966.75 16691075

[71] Eaton LA Kalichman SC Cain DN Cherry C Stearns HL Amaral CM. Serosorting sexual partners and risk for HIV among men who have sex with men. Am J Prevent Med. (2007) 33:479–85. 10.1016/j.amepre.2007.08.004 18022064 PMC3151147

[72] Crepaz N Marks G Liau A Mullins MM Aupont LW Marshall KJ. Prevalence of unprotected anal intercourse among HIV-diagnosed MSM in the United States: a meta-analysis. AIDS (2009) 23:1617–29. 10.1097/QAD.0b013e32832effae 19584704

[73] Steward WT Remien RH Higgins JA Dubrow R Pinkerton SD Sikkema KJ. Behavior change following diagnosis with acute/early HIV infection-a move to serosorting with other HIV-infected individuals. The NIMH multisite acute HIV infection study: III. AIDS Behav. (2009) 13:1054–60. 10.1007/s10461-009-9582-6 19504178 PMC2785897

[74] Snowden JM Raymond HF McFarland W. Prevalence of seroadaptive behaviours of men who have sex with men, San Francisco, 2004. Sex Trans Infect. (2009) 85:469–76. 10.1136/sti.2009.036269 19505875

[75] Jin F Crawford J Prestage GP Zablotska I Imrie J Kippax SC. Unprotected anal intercourse, risk reduction behaviours, and subsequent HIV infection in a cohort of homosexual men. AIDS (2009) 23:243–52. 10.1097/QAD.0b013e32831fb51a 19098494 PMC2768371

[76] Truong HHM Kellogg T Klausner JD Katz MH Dilley J Knapper K. Increases in sexually transmitted infections and sexual risk behaviour without a concurrent increase in HIV incidence among men who have sex with men in San Francisco: a suggestion of HIV serosorting? Sex Trans Infect. (2006) 82:461–6. 10.1136/sti.2006.019950 17151031 PMC2563862

[77] Snowden JM Raymond HF McFarland W. Seroadaptive behaviours among men who have sex with men in San Francisco: the situation in 2008. Sex Trans Infect. (2011) 87:162–4. 10.1136/sti.2010.042986 21076140

[78] Jablonski O Le Talec JY Seroadaptation Instead of Serosorting: A Broader Concept and a More Precise Process Model. Mexico City: XVII International AIDS Conference 3-8 August 2008 (2008).

[79] Cassels S Katz DA. Seroadaptation among men who have sex with men: emerging research themes. Curr HIV/AIDS Rep. (2013) 10:305–13. 10.1007/s11904-013-0188-2 24234489 PMC3946930

[80] Race KD. Revaluation of risk among gay men. AIDS Educ Prevent. (2003). 15:369–81. 10.1521/aeap.15.5.369.23822 14516021

[81] O'Leary A Guessing games: sex partner serostatus assumptions among HIV-positive gay and bisexual men. In: Halkitis PN, Gomez CA, Wolitski RJ, ediotrs. HIV+ sex: The Psychological and Interpersonal Dynamics of HIV-Seropositive Gay and Bisexual Men's Relationships. Washington, DC: American Psychological Association (2005). p. 121–132.

[82] MacKellar DA Valleroy LA Behel S Secura GM Bingham T Celentano DD. Unintentional HIV exposures from young men who have sex with men who disclose being HIV-negative. AIDS (2006) 20:1637–44. 10.1097/01.aids.0000238410.67700.d1 16868445

[83] Fernández-Dávila P Folch C Lorca KZ Casabona J Silence and assumptions: narratives on the disclosure of HIV status to casual sexual partners and serosorting in a group of gay men in Barcelona. Int J Sex Health (2011) 23:139–55. 10.1080/19317611.2011.574785

[84] O'Connell AA Reed SJ Serovich JA. The efficacy of serostatus disclosure for HIV transmission risk reduction. AIDS Behav. (2015) 19:283–90. 10.1007/s10461-014-0848-2 25164375 PMC4435532

[85] Golden MR Dombrowski JC Kerani RP Stekler JD. Failure of serosorting to protect African American men who have sex with men from HIV infection. Sex Trans Dis. (2012) 39:659–64. 10.1097/OLQ.0b013e31825727cb 22902660 PMC3424487

[86] Marks G Millett GA Bingham T Lauby J Murrill CS Stueve A. Prevalence and protective value of serosorting and strategic positioning among black and latino men who have sex with men. Sex Trans Dis. (2010) 37:325–7. 10.1097/OLQ.0b013e3181c95dac 20081556

[87] Wei C Raymond HF Guadamuz TE Stall R Colfax GN Snowden JM. Racial/Ethnic differences in seroadaptive and serodisclosure behaviors among men who have sex with men. AIDS Behav. (2010) 15:22–9. 10.1007/s10461-010-9683-2 20217468

[88] Wilton L Koblin B Nandi V Xu G Latkin C Seal D. Correlates of seroadaptation strategies among black men who have sex with men (MSM) in 4 US Cities. AIDS Behav. (2015) 19:2333–46. 10.1007/s10461-015-1190-z 26363789 PMC4638113

[89] Van den Boom W Stolte I Sandfort T Davidovich U Serosorting and sexual risk behaviour according to different casual partnership types among MSM: the study of one-night stands and sex buddies. AIDS Care (2012) 24:167–73. 10.1080/09540121.2011.603285 21861633 PMC3561689

[90] van den Boom W Konings R Davidovich U Sandfort T Prins M Stolte IG Is serosorting effective in reducing the risk of hiv infection among men who have sex with men with casual sex partners? J Acquir Immune Defic Syndr. (2014) 65:375 10.1097/QAI.0000000000000051 24189150 PMC3947546

[91] Pinkerton SD. Acute HIV infection increases the dangers of serosorting. Am J Prevent Med. (2008) 35:184. 10.1016/j.amepre.2008.04.012 18617087 PMC2539063

[92] Cairns G New Directions in HIV Prevention: Serosorting and Universal Testing. IAPAC Monthly, 12, 42–5 (2006). Available online at: http://www.ncbi.nlm.nih.gov/pubmed/17249137 17249137

[93] Katz DA Golden MR Stekler JD. Use of a home-use test to diagnose HIV infection in a sex partner: a case report. BMC Research Notes (2012) 5:440. 10.1186/1756-0500-5-440 22894746 PMC3598546

[94] Carballo-Diéguez A Frasca T Dolezal C Balan I. Will gay and bisexually active men at high risk of infection use over-the-counter rapid HIV tests to screen sexual partners? J Sex Res. (2012) 49:379–87. 10.1080/00224499.2011.647117 22293029 PMC3600862

[95] Golub SA Tomassilli JC Parsons JT. Partner serostatus and disclosure stigma: Implications for physical and mental health outcomes among HIV-positive adults. AIDS Behav. (2009) 13:1233–40. 10.1007/s10461-008-9466-1 18843532

[96] Koester K Amico RK Gilmore H Liu A McMahan V Mayer K. Risk, safety and sex among male PrEP users: time for a new understanding. Cult Health Sex. (2017) 19:1301–13. 10.1080/13691058.2017.1310927 28415911

[97] Suarez T Miller J. Negotiating risks in context : a perspective on unprotected anal intercourse and barebacking among men who have sex with men — where do we go from here? Arch Sex Behav. (2001) 30:287–300. 10.1023/A:1002700130455 11330118

[98] Mykhalovskiy E Rosengarten M Editorial: HIV/AIDS in its third decade: renewed critique in social and cultural analysis - an introduction. Soc Theory Health (2009) 7:187–95. 10.1057/sth.2009.13

[99] Rosengarten M HIV interventions: biomedicine and the traffic between information and flesh (In vivo: the cultural mediations of biomedical science). Seattle, WA: University of Washington Press (2010). 10.1080/09505431.2012.691470

[100] Braine N van Sluytman L Acker C Friedman S Des Jarlais DC. Sexual contexts and the process of risk reduction. Culture Health Sex. (2011) 13:797–814. 10.1080/13691058.2011.582688 21656412

[101] Grace D Chown SA Jollimore J Parry R Kwag M HIV-negative gay men' s accounts of using context-dependent sero- adaptive strategies. Cult Health Sex. (2014) 16:316–30. 10.1080/13691058.2014.883644 24571102

[102] Bourne A Dodds C Keogh P Weatherburn P Non-condom related strategies to reduce the risk of HIV transmission: perspectives and experiences of gay men with diagnosed HIV. J Health Psychol. (2015). 21:2562–71. 10.1177/1359105315581066 25947230

[103] Holt M Lea T Mao L Zablotska I Prestage G De Wit J. HIV prevention by Australian gay and bisexual men with casual partners: the emergence of undetectable viral load as one of a range of risk reduction strategies. J Acq Immune Defic Syndr. (2015) 70:545–8. 10.1097/QAI.0000000000000787 26258572

[104] Jin F Prestage GP Mao L Mary Poynten I Templeton DJ Grulich AE “Any condomless anal intercourse” is no longer an accurate measure of HIV sexual risk behavior in gay and other men who have sex with men. Front Immunol. (2015) 6:86 10.3389/fimmu.2015.00086 25774158 PMC4343002

[105] Persson A “The world has changed”: pharmaceutical citizenship and the reimagining of serodiscordant sexuality among couples with mixed HIV status in Australia. Soc Health Illness (2016) 38:380–95. 10.1111/1467-9566.12347 26360799

[106] CHRP East Bay AIDS Center: CRUSH: Connecting Resources For Urban Sexual Health (2012). Available online at: http://www.californiaaidsresearch.org/files/award-abstracts/prevention-and-linkage-to-care/crush.html

[107] Burack J Grant RM Myers JJ Connecting Resources for Urban Sexual Health (CRUSH). ClinicalTrials.gov [Internet]. Identifier NCT02183909. Bethesda (MD): National Library of Medicine (US) (2017). Available online at: https://clinicaltrials.gov/show/NCT02183909

[108] Ritchie J Spencer L Qualitative data analysis for applied policy research. Anal Qual Data (1994) 25:173–94. 10.4135/9781412986274

[109] Ryan GW Bernard HR Techniques to identify themes. Field Methods (2003) 15:85–109. 10.1177/1525822x02239569

[110] Grossman H Anderson P Grant R Gandhi M Mohri H Markowitz M (2016). Newly Acquired HIV-1 Infection with Multi-Drug Resistant (MDR) HIV-1 in a Patient on TDF/FTC-based PrEP. HIV Research for Prevention (HIVR4P) 2016 conference, Chicago, October 2016, abstract OA03.06LB. Available online at: http://webcasts.hivr4p.org/console/player/33033?mediaType=slideVideo&

[111] Donnell D Ramos E Celum C Baeten J Dragavon J Tappero J (2016). The Effect of Oral Pre-exposure Prophylaxis on the Progression of HIV-1 Seroconversion. HIV Research for Prevention (HIVR4P) 2016 Conference, Chicago, October 2016, Abstract OA03.01. http://webcasts.hivr4p.org/console/player/33028?mediaType=audio&

[112] Hoornenborg E Prins M Achterbergh RCA Woittiez LR Cornelissen M Jurriaans S. Acquisition of wild-type HIV-1 infection in a patient on pre-exposure prophylaxis with high intracellular concentrations of tenofovir diphosphate: a case report. Lancet HIV (2017) 4:e522–8. 10.1016/S2352-3018(17)30132-7 28919303

[113] Holt M Lea T Mao L Kolstee J Zablotska I Duck T. Community-level changes in condom use and uptake of HIV pre-exposure prophylaxis by gay and bisexual men in Melbourne and Sydney, Australia: results of repeated behavioural surveillance in 2013-17. Lancet HIV (2018) 5:Pe448–56. 10.1016/S2352-3018(18)30072-9 29885813

